# A matter of months: High precision migration chronology of a Bronze Age female

**DOI:** 10.1371/journal.pone.0178834

**Published:** 2017-06-05

**Authors:** Karin Margarita Frei, Chiara Villa, Marie Louise Jørkov, Morten E. Allentoft, Flemming Kaul, Per Ethelberg, Samantha S. Reiter, Andrew S. Wilson, Michelle Taube, Jesper Olsen, Niels Lynnerup, Eske Willerslev, Kristian Kristiansen, Robert Frei

**Affiliations:** 1 National Museum of Denmark, Department of Conservation and Natural Sciences, Environmental Archaeology and Material Science, Kongens Lyngby, Denmark; 2 University of Copenhagen, Department of Forensic Medicine, Laboratory of Biological Anthropology, Copenhagen, Denmark; 3 Centre for GeoGenetics, Natural History Museum of Denmark, University of Copenhagen, Copenhagen, Denmark; 4 National Museum of Denmark, Department of Research and Exhibition, Ancient Cultures of Denmark and the Mediterranean, Copenhagen, Denmark; 5 Museum Sønderjylland, Archaeology, Haderslev, Denmark; 6 School of Archaeological Sciences, University of Bradford, Bradford, United Kingdom; 7 University of Aarhus, Department of Physics and Astronomy, Aarhus, Denmark; 8 Institute for Historical Studies, University of Gothenburg, Gothenburg, Sweden; 9 Department of Geoscience and Natural Resource Management, University of Copenhagen, Copenhagen, Denmark; University of Florence, ITALY

## Abstract

Establishing the age at which prehistoric individuals move away from their childhood residential location holds crucial information about the socio dynamics and mobility patterns in ancient societies. We present a novel combination of strontium isotope analyses performed on the over 3000 year old “Skrydstrup Woman” from Denmark, for whom we compiled a highly detailed month-scale model of her migration timeline. When combined with physical anthropological analyses this timeline can be related to the chronological age at which the residential location changed. We conducted a series of high-resolution strontium isotope analyses of hard and soft human tissues and combined these with anthropological investigations including CT-scanning and 3D visualizations. The Skrydstrup Woman lived during a pan-European period characterized by technical innovation and great social transformations stimulated by long-distance connections; consequently she represents an important part of both Danish and European prehistory. Our multidisciplinary study involves complementary biochemical, biomolecular and microscopy analyses of her scalp hair. Our results reveal that the Skrydstrup Woman was between 17–18 years old when she died, and that she moved from her place of origin -outside present day Denmark- to the Skrydstrup area in Denmark 47 to 42 months before she died. Hence, she was between 13 to 14 years old when she migrated to and resided in the area around Skrydstrup for the rest of her life. From an archaeological standpoint, this one-time and one-way movement of an elite female during the possible “age of marriageability” might suggest that she migrated with the aim of establishing an alliance between chiefdoms. Consequently, this detailed multidisciplinary investigation provides a novel tool to reconstruct high resolution chronology of individual mobility with the perspective of studying complex patterns of social and economic interaction in prehistory.

## Introduction

Ancient human migration studies are currently witnessing a knowledge-revolution brought about mainly by contributions from ancient DNA (aDNA) and strontium isotope analyses [1]. It has recently become clear that European prehistory saw several major migratory events that transformed the continent genetically and culturally (e.g., [2,3]). While DNA analyses have provided this new evidence for migrations on a larger chronological and geographical scale, strontium isotope analyses have yielded information on the migration of single individuals allowing for the identification of migrants at specific sites [4–8]. Recently, new methodological developments in strontium isotope analyses based on soft human tissues such as scalp hair and fingernails have allowed scientists to trace the mobility of single individuals in unprecedented detail, providing the possibility of identifying travel and movement on a month-to-month scale [5,9]. Consequently, when conducting socio-dynamic studies of ancient societies, strontium isotope analyses have the advantage of being able to map the life-movements of single individuals within the prehistoric landscape, thereby providing essential information for the reconstruction of specific interaction patterns.

**Fig 1:**
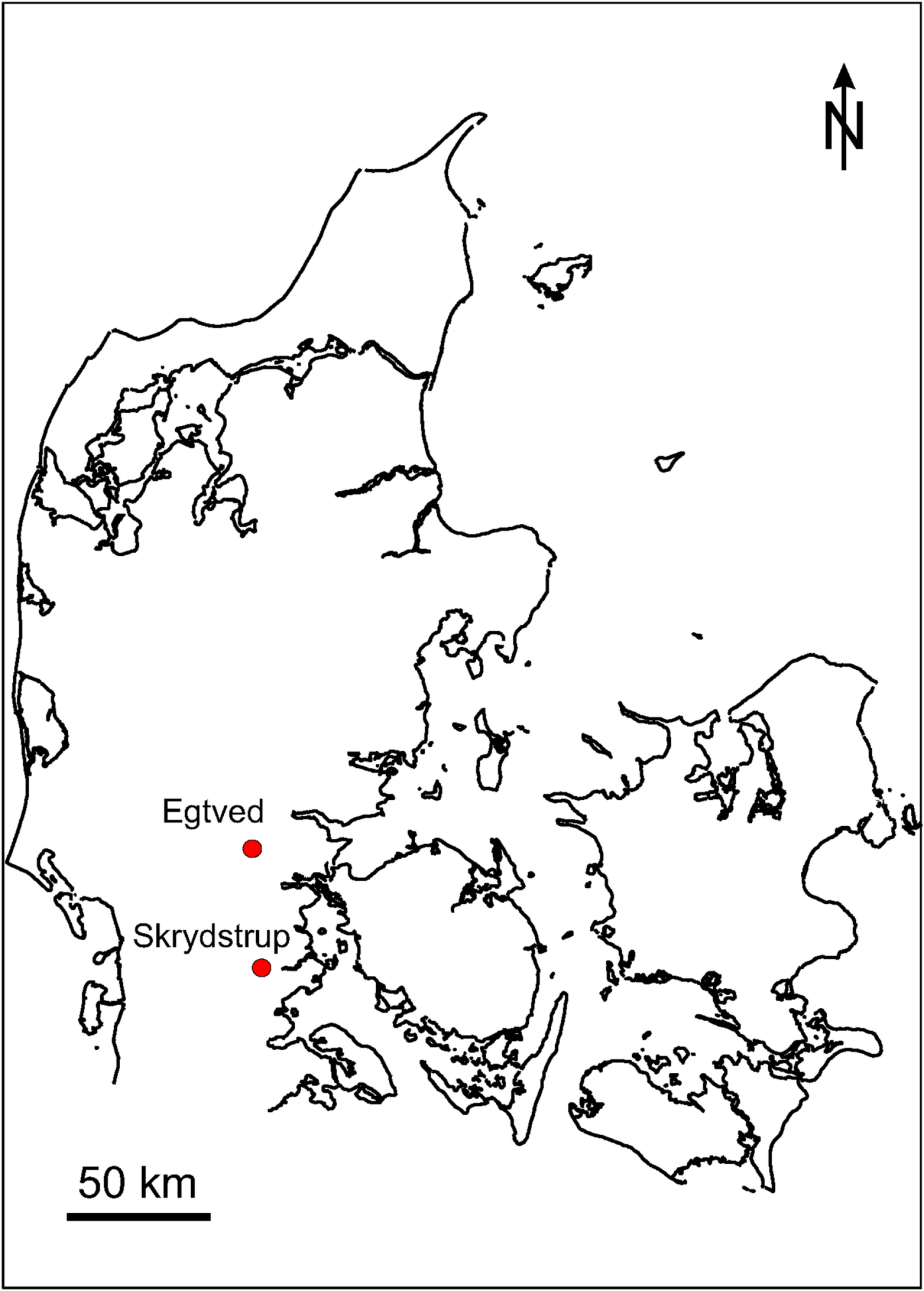
Map of Denmark indicating the Skrydstrup and the Egtved sites.

**Fig 2:**
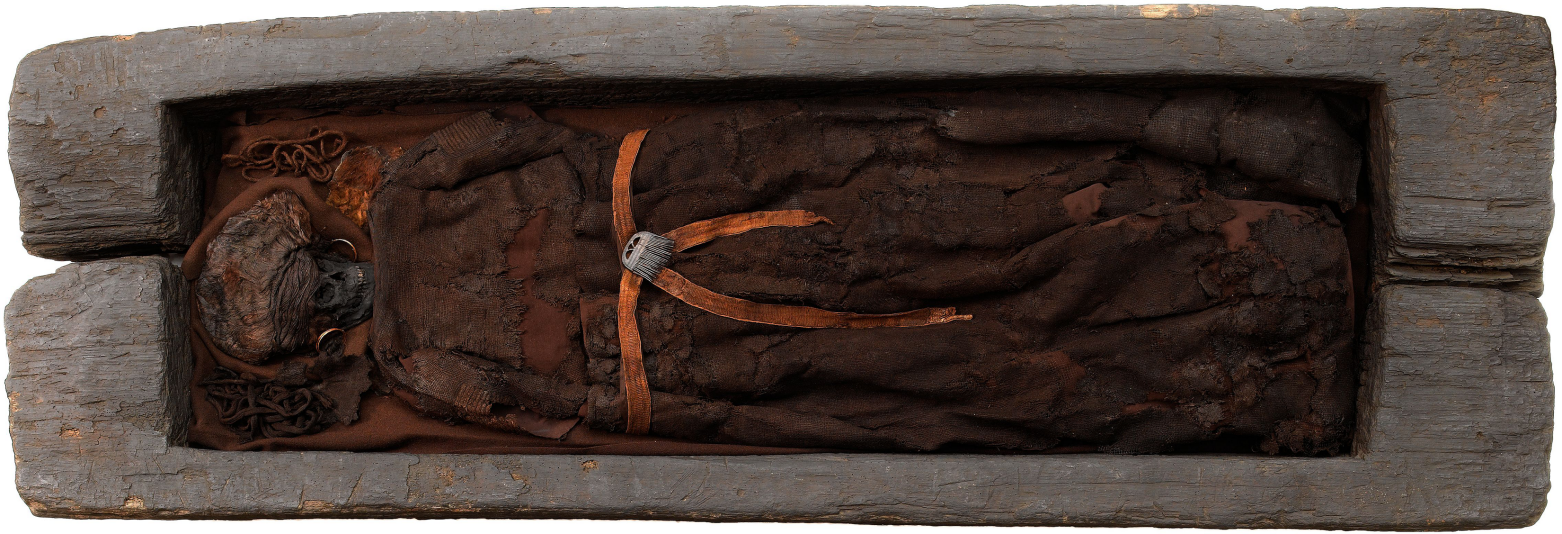
The Skrydstrup Woman as she is exhibited today at the National Museum of Denmark (Photo: Courtesy of The National Museum of Denmark).

We conducted a combination of high-resolution strontium isotope analyses with anthropological investigations including CT scanning and 3D visualizations on the remains of an elite female individual known as the Skrydstrup Woman dating to more than 3000 years ago, with the aim of mapping her movements throughout different periods of her life. The Skrydstrup Woman was unearthed in 1935 from a burial mound in southern Denmark (Figs 1 and 2, and S1 Text) [10]. In addition to those anthropological and strontium isotope analyses, we conducted a series of scientific analyses on her scalp hair, including biomolecular, light stable isotope and microscopic analyses.

The Skrydstrup Woman dates to the Nordic Early Bronze Age (1700–1100 BC), more specifically to the period III, 1300–1100 BC (S1 Text). The Early Bronze Age was a pan-European period characterized by technical innovation and social transformations stimulated by long-distance connections [11] triggered by the distribution of metals such as copper and tin for bronze production [12–14]. Therefore this period is sometimes referred to as “The First Golden Age”, and it exhibited cultural traits that were shared in many parts of Europe. Nevertheless, many key questions remain unanswered [15]. One of these -itself central to understanding Bronze Age society- is directly related to how long-distance trade was organized and maintained. Exotic artifacts found across Northern Europe indicate extensive connections with distant areas [11,16,17]. Thus, recent chemical studies of glass beads from Bronze Age female graves in Denmark confirm the existence of contacts between Egypt, Mesopotamia and Denmark during the 14th -12th century BC [18,19]. It is our objective to investigate how networks such as these were created and we aim at the exploring the possibilities to identify at what age people migrated, how far they travelled, and how mobile they were after reaching their destination.

In Scandinavia, the Nordic Bronze Age (1700–500 BC) left strong cultural imprints upon the landscape, features that include the ten thousands of burials mounds and the thousands of rock carvings along the Scandinavian coastline [20]. In addition, Denmark possesses a unique collection of exceptionally well-preserved human remains: the well-known oak-coffin people [21,22]. These individuals, both men and women, represented the elite of free farmers and warriors during the Early Bronze Age, and are therefore key to an understanding of how international trade networks were established and maintained [12]. The Skrydstrup Woman belongs to this group of oak-coffin burials.

## Materials and methods

### The Skrydstrup Woman

The Skrydstrup Woman was unearthed in 1935 from a burial mound in southern Denmark [10]. This type of burial mound was constructed of enormous amounts of turf layers laid with the grassy side down, and surrounded by a wall of stones around the mound’s base [23]. Inside the core of some of the Bronze Age mounds anoxic waterlogged conditions were created by the formation of ferrous layers which sealed the mound and thus prevented decomposition of organic remains [23]. The Skrydstrup Woman’s mound was part of wider group which consisted of a total of eight burial mounds [24,25]. The Skrydstrup Woman’s remains represent the mound’s primary grave and consist of skeletal as well as soft tissues (S1 Text). The latter include parts of her cheeks and chin, eyebrows, eyelids, eyelashes and her long hair [10,21]. Of these, only the hair still remains. Measuring over 60 centimeters, Skrydstrup Woman’s hair is set in a highly complex hairstyle very unusual to the area in which she was found [10] (S1 Text). Only in northwestern Denmark another find in Karby showed a similarly complex hair style [26]. Of the Skrydstrup Woman skeletal tissues, a large part still remains; her teeth especially are well preserved [10].

In common with other oak coffin burials, the Skrydstrup Woman rested on an ox-hide and was wearing a short-sleeved wool blouse decorated with embroidery on the sleeves and at the neckline. She was also equipped with a large square wool textile piece gathered at the top by a belt which covered her from waist to ankle [10] (Fig 2). Additionally, she had a wool cap, a horn comb attached to her belt and a large set of gold spiral rings which lay by her ears (S1 Text) [10].

### Anthropological analyses

#### Previous analyses

**Fig 3:**
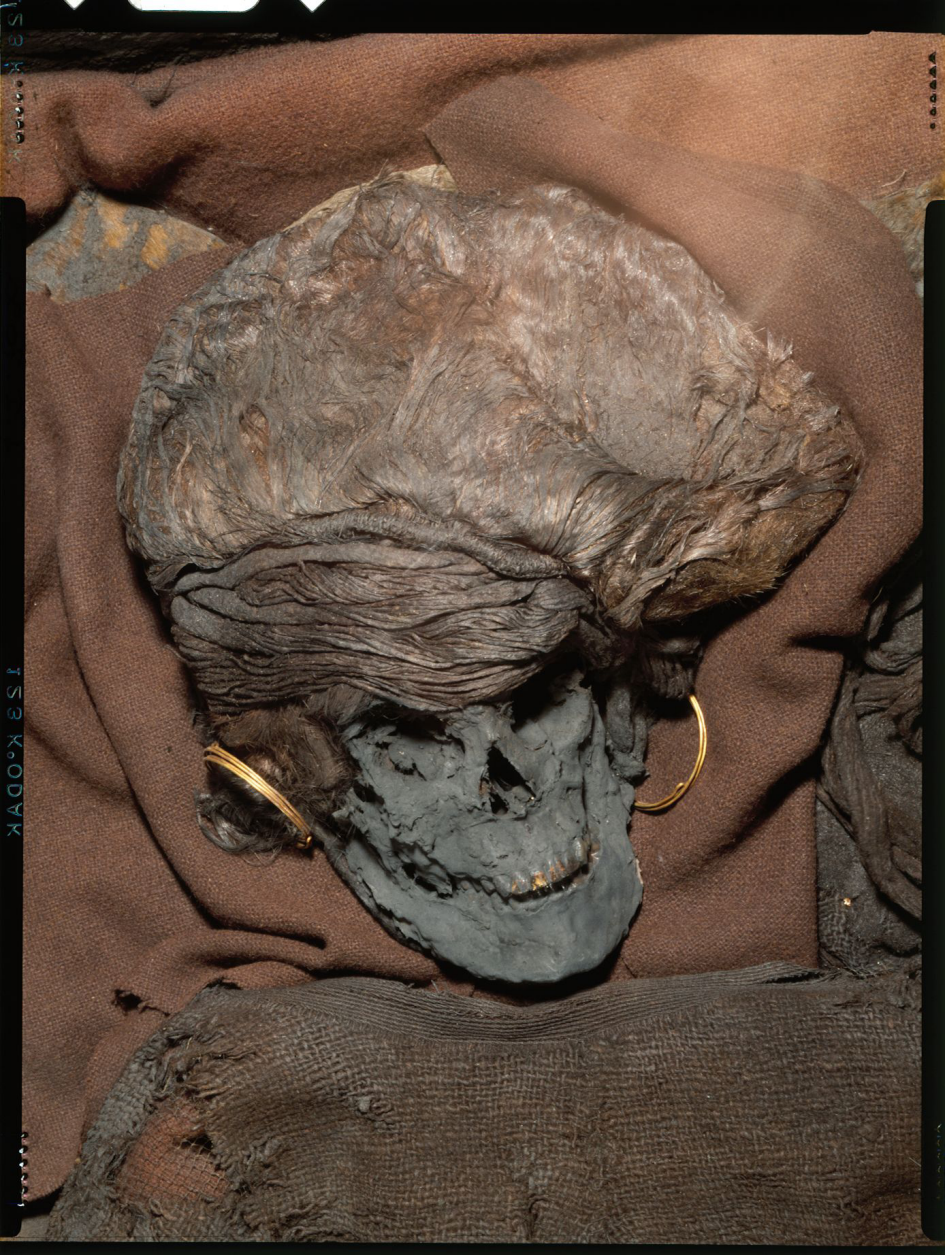
A close up image of the Skrydstrup Woman’s facial remains (Photo: Lennart Larsen, National Museum of Denmark).

The first anthropological analyses of the human remains from the Skrydstrup Woman were made in 1939 by Dr. Fischer-Møller among others [21]. When discovered the remains were brittle and fragile. Glycerin was applied to the bones and hair as a conservation measure in an attempt to preserve them, leaving a thick dark coating on the remains which still is visible today (Fig 3). However, the bones were likely already blackened through the combined action of humic and tannic acids within the core of the burial mound and oak coffin, an observation previously made in similar acidic environments. These conditions are similar to those involved in tanning and associated with the Maillard reactions known from bog bodies [23,27]. Initial odontological investigations showed that all teeth were visible with the exception of the wisdom teeth. The latter were not yet fully developed in the maxilla and had not erupted in the mandible. The teeth are in excellent condition and present no evidence of dental caries or other disease [21]. Based on these initial analyses, Dr. Fischer-Møller concluded that the human remains belonged to a young female whose chronological age was estimated to be roughly 20 years at most.

#### Present analyses

**Fig 4:**
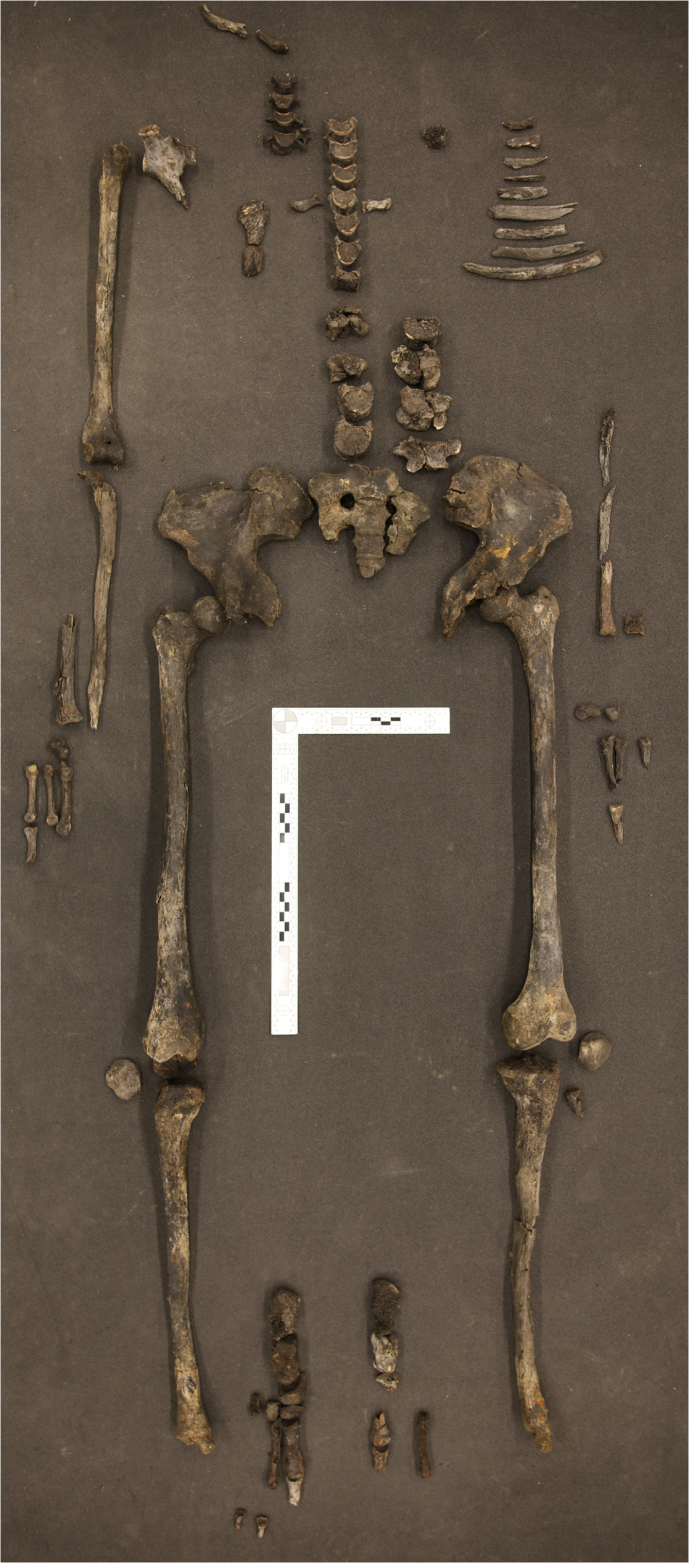
Overview of the postcranial skeleton.

**Fig 5:**
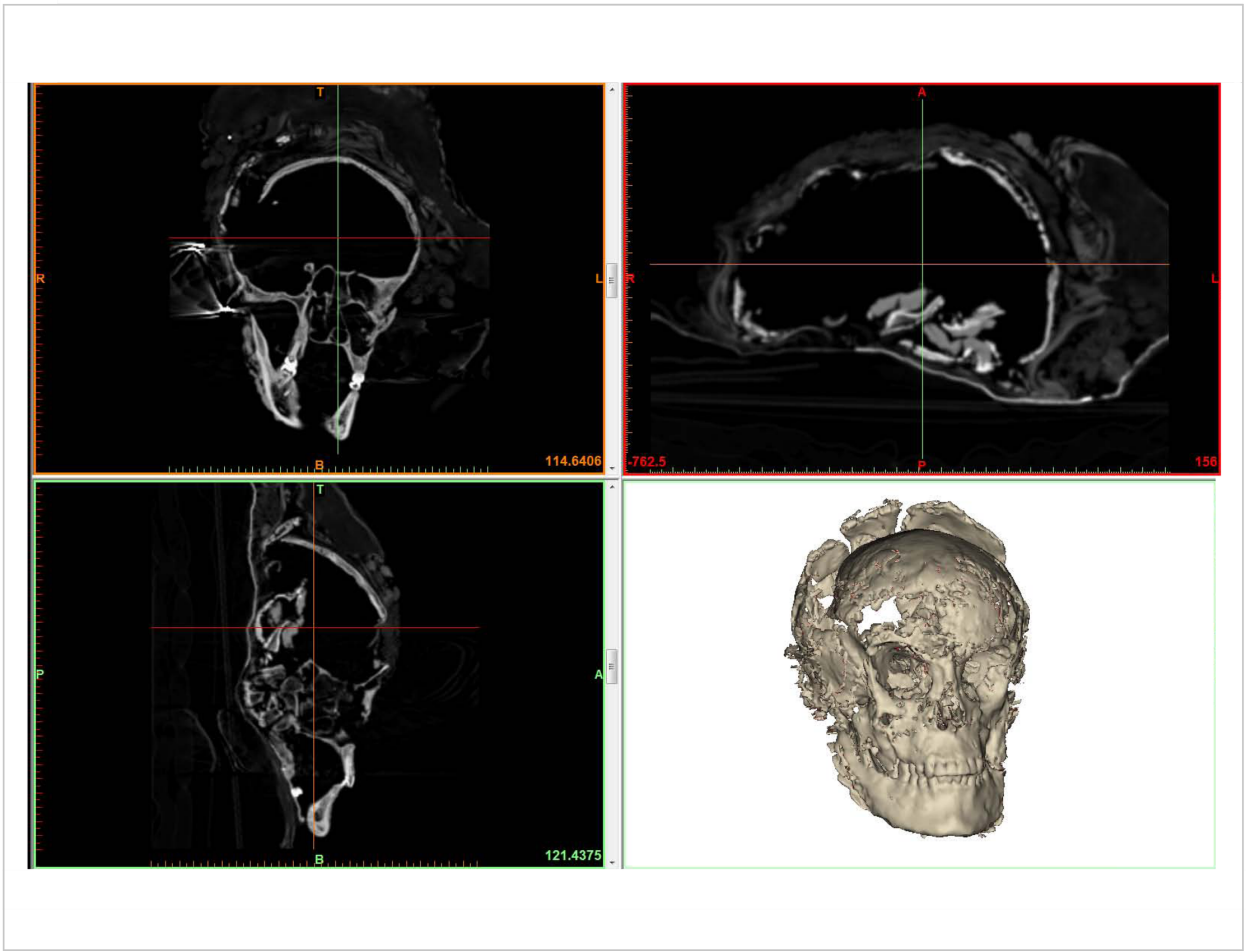
CT images (axial, coronal, and sagittal views) and 3D visualization of the skull.

Our anthropological investigations presented herein were carried out based on the evaluation of the osteological material (Fig 4) as well as CT images (Fig 5) and 3D visualizations. Sex determination was based on the morphological features of the *os coxae*, *sacrum*, the maximum diameter of the femoral head and the appearance of the long bones according to standard anthropological techniques (e.g. [28]). The Skrydstrup Woman’s age at death was estimated based on the status of the epiphyseal closure of the long bones, the pelvic girdle and vertebral rims ([29] and references therein) as well as on the development and eruption of the teeth [30]. Stature was estimated using the methods developed by Ruff et al. [31].

### Strontium isotope analyses

#### Human tooth enamel

We conducted strontium isotope analyses on the tooth enamel from one of the first molars and one of the third molars (a wisdom tooth) of the Skrydstrup Woman. Tooth enamel in humans is, unlike tooth dentine, resistant to diagenesis and therefore often used in archaeological investigations to trace human migration [32,33]. The enamel from the first molar begins to form *in utero* and finalizes mineralization at c. three years of age, thus providing information about the place of an individual’s childhood origin [32,33]. In contrast, the crown of the third molar is more variable and represents a longer period of time from the early adolescent years up to approximately 16 years of age [34]. We sampled both these teeth in order to create a long-term timeline related to the places in which the Skrydstrup Woman had lived throughout her life.

Prior to sampling, the surface of the enamel of the loose third molar was burred off with a dental diamond drill (including the glycerin layer) and a clean piece of the third molar enamel (with no dentine) was sampled. The first molar was sampled *in situ* (while still in the skull). Following the pre-cleaning procedure and removal of the enamel’s surface, a few milligrams of enamel powder remained. The two enamel samples were dissolved in 7 ml Teflon beakers (Savillex™) in a 1:1 solution of 0.5 ml 6 N HCl (Seastar) and 0.5 ml 30% H_2_O_2_ (Seastar). The samples dissolved within 5 minutes, after which the solutions were dried on a hotplate at 80°C. Subsequently, the enamel samples were taken up in a few drops of 3N HNO_3_ and then loaded onto disposable 100 μl pipette tip extraction columns into which we fitted a frit which retained a 0.2 ml stem volume of intensively pre-cleaned mesh 50–100 SrSpec™ (Eichrome Inc.) chromatographic resin. The elution recipe essentially followed that by Horwitz et al. [35], albeit scaled to our needs in so far as strontium was eluted / stripped by pure deionized water and then the eluate dried on a hotplate.

Thermal ionization mass spectrometry was used to determine the Sr isotope ratios. Samples were dissolved in 2.5 μl of a Ta_2_O_5_-H_3_PO_4_-HF activator solution and directly loaded onto previously outgassed 99.98% single rhenium filaments. Samples were measured at 1250–1300°C in dynamic multi-collection mode on a VG Sector 54 IT mass spectrometer equipped with eight faraday detectors (Institute of Geosciences and Natural Resource Management, University of Copenhagen). Five nanogram loads of the NBS 987 Sr standard that we ran during the time of the project yielded ^87^Sr/^86^Sr = 0.710241 +/- 0.000011 (n = 5, 2σ).

#### Human scalp hair

As previously mentioned, glycerin had been applied to the head and scalp hair for conservation purposes shortly after the Skrydstrup Woman was found. In order to remove this layer, we submerged the hair segments in a 0.01N Na(OH) bath and subsequently rinsed it with ultrapure (Milli-Q™; Millipore Corporation) H_2_O. We conducted a series of effective acid leaching steps for pre-cleaning purposes, including a mild acid 0.1 M HCl bath [5,36,37], a rinse in MQ H_2_O, and finally a submerge in 20% hydrofluoric acid (HF). This last step effectively removed both solid micro-particles as well as the lipid portion of hair known to be sensitive to environmental contamination [5,38,39]. Lastly, after another rinsing step with MQ H_2_O, the deeply pre-cleaned final residual hair portions, containing the nutritional strontium isotope information, were dissolved in a 1:1 solution of 30% HNO_3_ (Seastar™) and 30% H_2_O_2_ (Seastar™). These pre-cleaning steps have previously shown to effectively remove potential contamination after long time burial in order to recover the strontium nutritional fraction of the hair necessary to investigate mobility [5]. The samples tended to dissolve within 30 minutes. After decomposition, the solutions were dried on a hotplate at c. 80°C. Subsequently, the samples were taken up and analyzed following the same procedures as previously detailed above for tooth enamel.

Since modern human scalp hair grows an average of c. 1 cm per month, it is an archive which can potentially provide high-resolution diachronic information of diet and mobility when investigated by respective multi-disciplinary tracers [36,37,40,41]. Due to the complexity of hair style and the unique archaeological nature of the Skrydstrup Woman, the sampling amount needed to perform reliable strontium isotope analyses had to be balanced against the conservation needs to safeguard this important assemblage. For the strontium isotope investigation of the hair, we divided a 42.5 cm long hair shaft into 17 segments covering a total growth period of at least 51 months prior to death (this also includes a small sampling gap of c. 8 cm necessitated by the complicated hair style, see Table 1).

**Table 1:** Strontium isotope ratios of tooth enamel and hair from the Skrydstrup Woman.

Hair (Segment description)	Lenght of segment (cm)	Distance from scalp	weight (mg)	^87^Sr/^86^Sr	2σ (abs)
A (part 1)	3	c. 0.5–3.5 cm	16.18	**0.70871**	0.00004
B (part 1)	3.5	3.5–7 cm	14.27	**0.70873**	0.00004
C (part 1)	3	7–10 cm	23.07	**0.70918**	0.00004
D (part 1)	5.5	10–15.5	20.51	**0.70900**	0.00005
E (part lacking)	8	*gap of c*. *8 cm*	n.a.		
F (part 2)	2	23.5–25.5 cm	17.94	**0.70883**	0.00003
G (part 2)	2	25.5–27.5 cm	24.96	**0.70939**	0.00002
H (part 2)	2.5	27.5–30 cm	25.66	**0.71019**	0.00002
I (part 2)	3	30–33 cm	31.26	**0.70864**	0.00002
J (part 2)	1.5	33–34.5 cm	21.93	**0.70877**	0.00003
K (part 2)	1.5	34.5–36 cm (partial overlap)	21.07	**0.70923**	0.00002
L (part 3)	2.5	35–37.5 cm (partial overlap)	36.89	**0.70945**	0.00002
M (part 3)	2	37.5–39.5 cm	24.88	**0.70868**	0.00006
N (part 3)	2.5	39.5–42 cm	22.41	**0.70856**	0.00006
O (part 3)	3	42–45 cm	27.04	**0.70935**	0.00006
P (part 3)	2	45–47 cm	26.17	**0.71383**	0.00003
Q (part 3)	2	47–49 cm	31.01	**0.71347**	0.00003
R (part 3)	2	49–51 cm	24.46	**0.71354**	0.00002
*Total hair length analyzed*		*42*.*5 cm*			
*Estimated total hair length*		*60 cm*			
**Tooth enamel samples**					
Skrydstrup Woman	M3			**0.71326**	0.00003
Skrydstrup Woman	M1			**0.71375**	0.00004

#### Baseline

In order to obtain provenance information based on the strontium isotope system, the baseline or isoscape ranges of the isotopic composition of local bioavailable strontium must be known. Most of Denmark (hereafter defined as territorial Denmark) consists of a pre-Quaternary geological basement primarily composed of Tertiary and Cretaceous sediments (predominantly carbonates), all characterized by relatively low strontium isotopic compositions. These carbonates are overlain by glaciogenic sediments deposited during the last Quarternary Ice Ages. The baseline for territorial Denmark has previously been characterized and strontium isotope signatures range from ^87^Sr/^86^Sr = 0.708 to 0.711 [42–44]. Only the island of Bornholm (located south of Sweden in the Baltic Sea) has elevated bioavailable strontium isotope signatures (^87^Sr/^86^Sr > 0.711) due to the Precambrian basement which dominates most of the island [45]. Furthermore, in the present study we characterized the local bioavailable strontium isotope range in detail by measuring a total of 20 soil, plant and surface water samples from the area around Skrydstrup, including the area of the nearby contemporaneous Bronze Age settlement. We obtained ^87^Sr/^86^Sr values ranging from 0.70844 to 0.71069 (Table 2). For the baseline measurements mentioned above, we followed the procedures described in [45]

**Table 2:** Soil, plant leave and surface water baseline values.

Sample nr.	Sample type	^87^Sr/^86^Sr	2σ (abs.)
1	Soil	**0.71057**	0.00001
2	Plant leaves	**0.70961**	0.00002
3	Soil	**0.70909**	0.00001
4	Plant leaves	**0.70992**	0.00001
5	Soil	**0.70973**	0.00001
6	Soil	**0.71069**	0.00001
7	Plant leaves	**0.71006**	0.00001
8	Soil	**0.70849**	0.00001
9	Plant leaves	**0.70844**	0.00001
10	Soil	**0.70893**	0.00001
11	Plant leaves	**0.70875**	0.00001
12	Soil	**0.70889**	0.00001
13	Plant leaves	**0.70845**	0.00001
14	Water	**0.71002**	0.00001
15	Plant leaves	**0.71049**	0.00001
16	Soil	**0.71040**	0.00001
17	Water	**0.70890**	0.00001
18	Soil	**0.70909**	0.00001
19	Plant leaves	**0.71018**	0.00001
20	Plant leaves	**0.71008**	0.00001
Mean		0.70954	0.00153
Median		0.70967	
Lowest		0.70844	
Higest		0.71069	

### Additional scientific investigations of the scalp hair

#### Stable isotope analyses (δ^13^C and δ^15^N)

We conducted stable isotopes analyses of a 12 cm long scalp hair sample in order to provide some incremental data on the Skrydstrup Woman’s diet. The 12 cm long hair sample was cut into a total of 8 segments (each measuring roughly c. 1.5 cm) representing approximately the final 12 months of Skrydstrup Woman’s life.

Isotopic data based on δ^13^C and δ^15^N analyses varies predictably depending on dietary intake [46,47] and can, therefore, be used to distinguish between reliance on largely terrestrial-based or marine food resources in past populations as well as providing information about seasonal variation in diet and to potentially identify physiological impacts. Carbon stable light isotope ratios (δ^13^C) and nitrogen stable isotope ratios (δ^15^N) reflect the main sources of protein consumed at the time that the tissue formed. Nitrogen (δ^15^N) values increase as a result of metabolic fractionation by 2–5 ‰ at each trophic level of a food chain and can, therefore, provide information about the relative consumption of plant, animal and marine protein where sufficient baseline data exists [48].

The hair samples were prepared according to standard protocols [47] in which the adherent soil and exogenous organic deposits, including residues from historic conservation treatment with glycerol, were removed from the fiber surface by overnight soaking/ gentle agitation in organic solvent (2:1 methanol:chloroform) followed by sonication (3 x 15min). The organic solvent was then removed and the hair sample itself rinsed in deionized water (x3 separate washes, each with sonication). The final wash was decanted off and the cleaned sample was then frozen and lyophilized and preconditioned for weighing into tin capsules. The δ^13^C and δ^15^N ratios of the hair samples were measured by EA-IRMS using a ThermoFinnigan FlashEA 1112 elemental analyzer coupled to a DeltaPlus XL multicollector mass spectrometer at the University of Bradford Stable Light Isotope Facility. Each of the samples were analyzed as bulk samples (S1 Text).

#### Ancient DNA analyses

An ancient DNA (aDNA) extraction was attempted on a small subsample of hair (<10 mg), representing the residual sample from the Sr-analyses. Although we would normally consider such small amount to be insufficient for successful aDNA analysis, we decided to attempt this nonetheless with the wish to maximize analytical potential, given Skrydstrup Woman’s high scientific value. We used a highly sensitive silica-in-solution protocol, optimized for retaining short and degraded DNA molecules [3]. Using a Quibit Fluorometer, a very low (c. 100 ng/ml) DNA concentration was detected in the extract. Next, 20 μl of DNA extract was built into a double-stranded, blunt-end library using the NEBNext DNA Sample Prep Master Mix Set 2 (E6070) and Illumina specific adapters [49] with a PCR setup described previously [3].

#### Microscopic analyses

We investigated several strands of hair from close to the back of the Skrydstrup Woman’s neck plus a single fiber which represented a portion of the hair further from the scalp. The samples were all 5–10 cm long segments cut from longer strands. These hairs were examined with a stereo microscope and then a transmitted light microscope. Initial examination was conducted on non pre-treated hair which was not impeded by the surface contamination. For the transmitted light microscopy (TLM), the entire segment of hair was be arranged on a microscope slide under a large cover slip. Distilled water was used as the immersion medium. In addition, one piece of hair was embedded in polyethylene. Images were captured in plane polarized light using a neutral density filter.

#### ^14^C radiocarbon analyses

Previous ^14^C radiocarbon analyses made on a piece of untreated (not conserved) wool textile from the Skrydstrup find yielded an age of 2900 ± 80 BP (Lab. nr. K-3873/X-4586, National Museum of Denmark report A 6147, year 1983), placing the Skrydstrup burial within the Nordic Early Bronze Age Period III (1300–1100 BC). However, the Skrydstrup burial has traditionally been typologically dated to the earlier Period II (1500–1300 BC) of the Nordic Bronze Age, despite the fact that the grave is difficult to date archaeologically due to its lack of true typological markers [22,50,51]. This is complicated by the fact that the gold spiral rings may suggest a later date within Period III [22]. Due to these challenges, we performed new AMS ^14^C-analyses of the Skrydstrup Woman’s scalp hair, which revealed an age of 3009 ± 27 BP (Lab. nr. AAR: 25433 (S1 Text). Our ^14^C analyses correspond with the previous ^14^C analyses of the wool textile, placing the find within Nordic Early Bronze Age Period III (1300–1100 BC). The differences in the statistical ages of the two analyses can be due to the fact that the wool must be older than the Skrydstrup Woman, and could theoretically have been collected (cut from the sheep) quite some years before it was actually spun as a thread and weaved to a textile.

## Results and discussion

Ancient human mobility at the individual level is often studied by the application of radiogenic strontium isotopes to skeletal tissues [32,33]. When isotopic tracing and aDNA are applied on the same individual, important information on the socio-dynamics of ancient societies can be retrieved, such as in the case of the Stone Age human remains from Eulau in Germany [52]. The combined investigations of aDNA and strontium isotope analyses of these individuals which included men, women and children, revealed the earliest nuclear family to date with a specific type of social organization based on patrilocality and exogamy [52]. However, in studies where only skeletal tissues such as tooth enamel are analyzed, it is difficult and often impossible to estimate the age at which an individual actually moved to their final residential location and/or place of burial. The lack of this important information limits our understanding of the social dynamics of the periods under study.

New methodological developments in the field of strontium isotope analysis provide us with unprecedented opportunities to narrow down the time and, consequently, the timeframe for which these movements occurred. This type of detailed mobility investigations of single individuals is possible when hair and/or nails are also preserved [5,9]. Similarly, age determination can be more precise when several techniques are combined.

**Fig 6:**
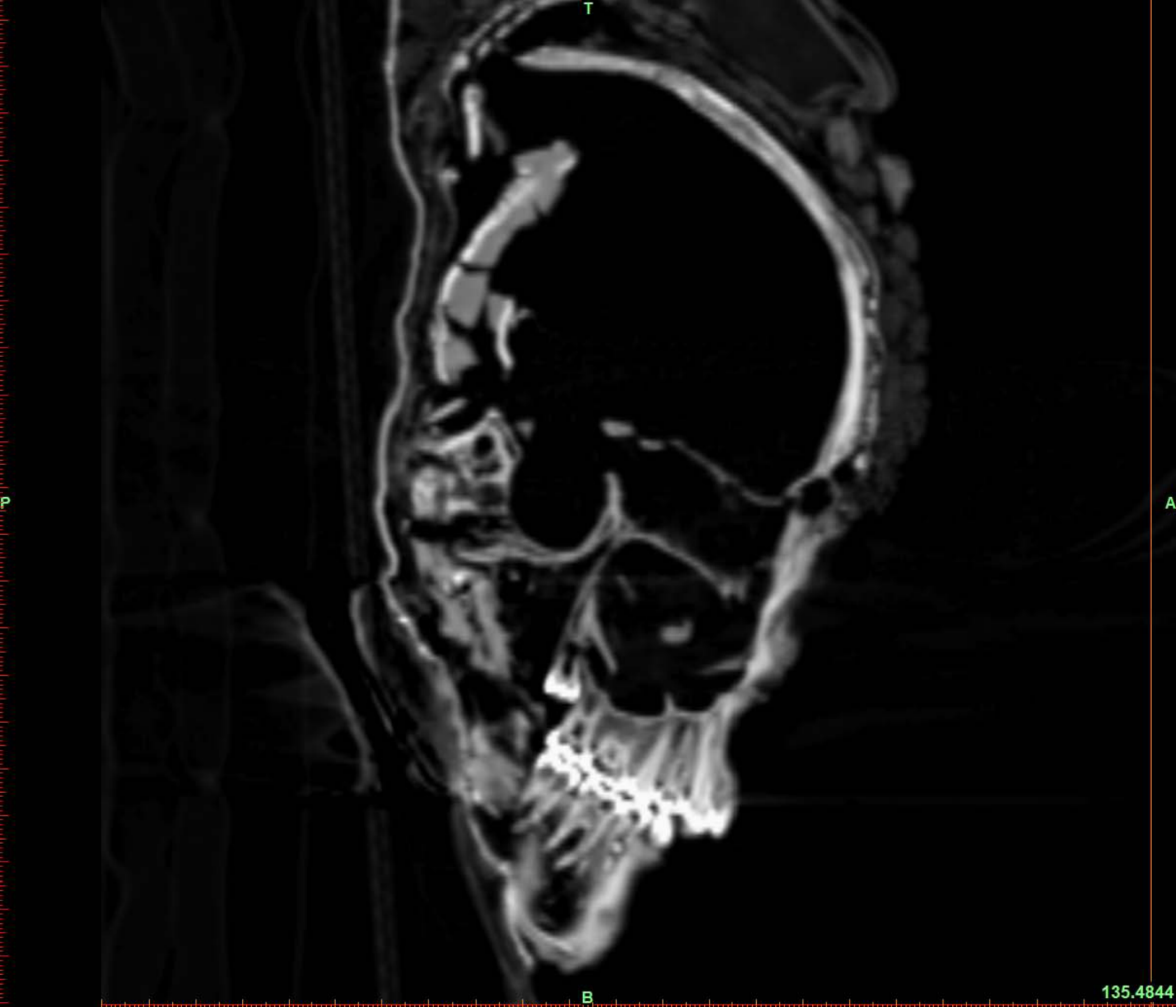
Sagittal CT view of the skull, showing the development state of the teeth.

Consequently, we estimated the age at death of the Skrydstrup Woman first by studying the status of epiphyseal closure of the long bones, the pelvic girdle and vertebral rims. These investigations rendered an estimated age at death of 17–18 years. Secondly, analysis of our CT-scans and 3D visualization revealed that the root of the left upper third molar was only partially formed, i.e. that only two thirds of the root was formed at the time of death (Fig 6). This feature indicates a maximum age of 17–18 years with both methodologies confirming the same age range for her estimated time of death. Furthermore, these findings were also in agreement with initial estimates of the Skrydstrup Woman’s maximum chronological age at death: 20 years [21].

Based on our present investigations of the morphological features of the *os coxae*, *sacrum*, the maximum diameter of the left femoral head (42.5mm) and the appearance of the long bones (being very gracile), we can confirm that the remains were those of a female individual.

There is ongoing debate as to which method should be applied in order to estimate the living stature from archaeological skeletal remains [53]. We applied an equation developed from Holocene European skeletal remains and compared the result with the initial measurements of the skeleton while still *in situ*. At excavation, the length of the Skrydstrup Woman was measured from the top of the skull to the heel at ca. 171.5–172 cm [10]. Our measurements (S1 Text) reveal that there is a difference between those initial measurements and the present anthropological measurements. This difference could be due to either measurement error or subsequent shrinkage of the bones over the last seven decades. Based on the maximum length of the femur we applied the Ruff et al. equation for females (32) which yielded a stature of 169.7cm±2.92cm. This result seems to fit the initial *in situ* measurements. We also compared the length and proportions of the femur to be able to compare it to other studies of Bronze Age populations from central Europe. These studies were conducted by Vančata and Charvátová [54] and showed that there was a marked variation in femur lengths and proportions for males and females. When these values are compared with the femur length of the Skrydstrup Woman, it becomes clear that her femur lengths lie closer to the mean femur length calculated for Bronze Age males (mean 442.2–445.3 cm) than to that computed for females (mean 403.1cm to 411.4 cm). This seems to indicate that the Skrydstrup Woman was very tall for her time. Despite their fragmented condition it is clear that the Skrydstrup Woman’s long bones are gracile and long.

Although there was no evidence of pathology, a congenital anomaly -a missing styloid of the third left metacarpal- was found. However, the latter would have had no impact on the Skrydstrup Woman’s health or daily activities.

**Fig 7:**
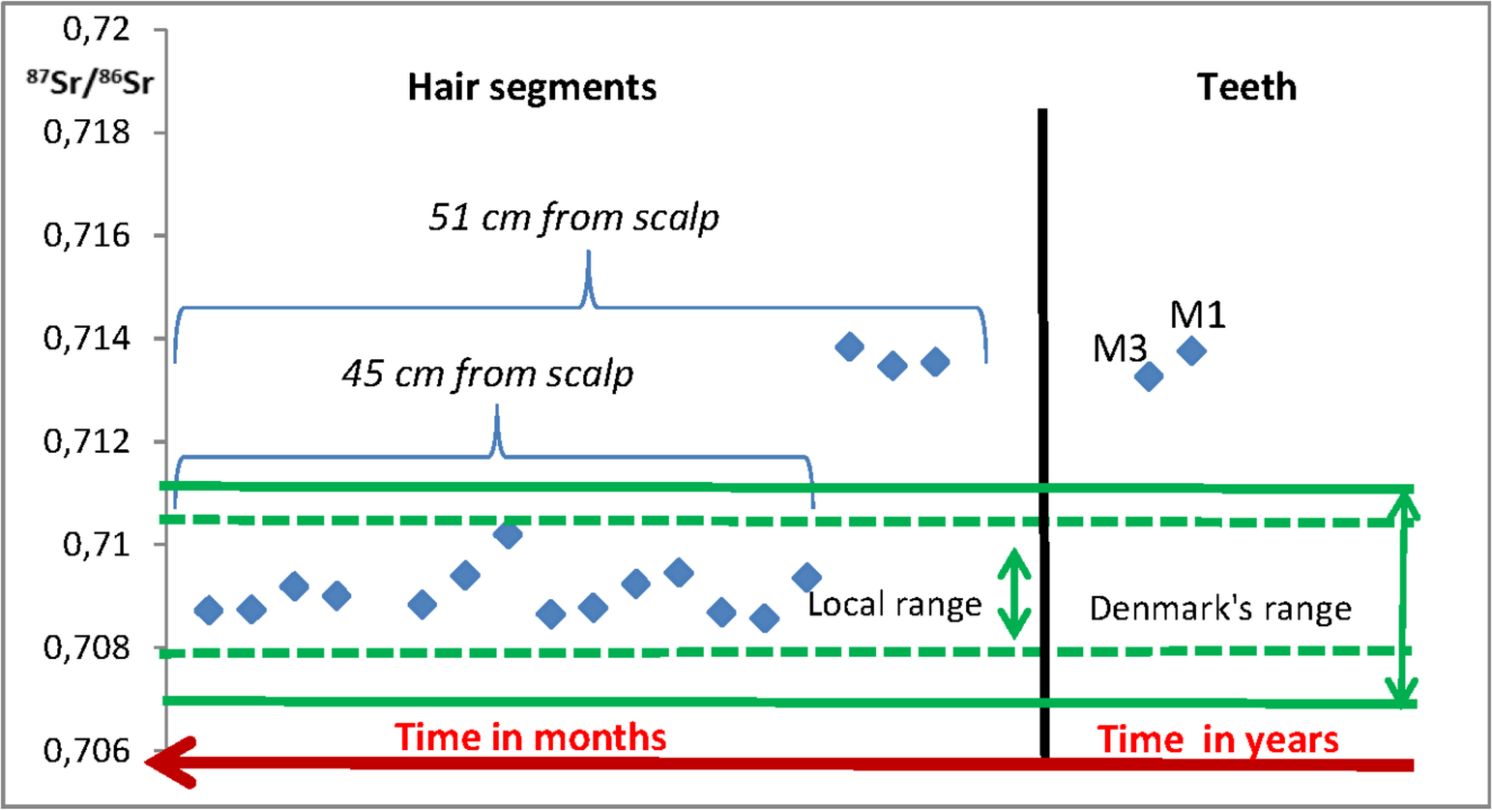
Strontium isotope diagram depicting the results of the human remains of the Skrydtsrup Woman (hair segments and tooth enamel). The area between the horizontal green dotted lines indicate the bioavailable strontium isotopic range of the area of Skrydstrup calculated from the values in Table 2. The area between the horizontal green solid lines indicate the bioavailable strontium isotopic range for territorial Denmark.

Strontium isotope analysis of the tooth enamel from Skrydstrup Woman’s first molar (M1) yield a value of ^87^Sr/^86^Sr = 0.71375 (± 0.00004; 2σ), which implies a nonlocal origin when put into context with her place of burial as well as to territorial Denmark in its entirety. Furthermore, the third molar (M3) also yield a nonlocal value of ^87^Sr/^86^Sr = 0.71326 (± 0.00003; 2σ), though not as radiogenic a value as the one obtained for M1. Both values imply that the Skydstrup Woman lived most of her life outside present-day territorial Denmark. In contrast, some of the scalp hair segments yield a local strontium isotope signature, while other hair segments have strontium isotope values similar to the elevated, nonlocal signatures recorded in the tooth enamel of M1 and M3. In general, the strontium isotope analyses of the scalp hair span a relatively large range of values, both local and nonlocal which range from ^87^Sr/^86^Sr = 0.70856 (± 0.00006; 2σ) to 0.71383 (± 0.00003; 2σ), (Table 1 and Fig 7). Interestingly, an abrupt change in the range of strontium isotope signatures in the hair occurs between segment O and P (Table 1). These particular hair segments represent a period c. 47 to 42 months prior to the Skrydstrup Woman’s death. We posit that this relatively short time period represents the months during which the Skrydstrup Woman travelled from a place outside of territorial Denmark to the Skrydstrup area. After this period, the strontium isotope signatures measured on the rest of the hair segments -representing a period of c. >40 months before death- all yield local signatures with a range from ^87^Sr/^86^Sr = 0.71019 (±0.00002) to 0.70856 (±0.00006). This range coincides with the site-specific local isotope range of bioavailable strontium that we measured in the samples from the Skrydstrup area (^87^Sr/^86^Sr = 0.71069 to 0.70844). We are well aware of the problematics around potential diagenetic effects, particularly of ancient human soft tissues, such as hair. However, as demonstrated in earlier studies [5,9,38,39], the herein applied pre-cleaning treatment is effective in removing non-nutritional strontium fractions from hair. Furthermore, in our present study the variations in strontium isotopic signatures measured on the hair segments of the Skrydstrup Woman, in fact point to a successful application of the in depth pre-cleaning treatment in that it implies an effective removal of contaminant strontium from the hair. Hence, the change in the trend of the strontium isotope data indicates that potential diagenetic effects can likely be ruled out, as with a substrate like hair one would expect this to be affected fairly uniformly in waterlogged conditions.

The strontium isotopic composition of the hair segments imply, that following migration to the Skrydstrup area, the Skydstrup Woman most probably resided locally for the remainder of her life. Finally, the lower strontium isotopic ratio in the third molar as compared to the first molar suggests some kind of change in the local baseline range, perhaps related to movement or variation of food sourcing. Another alternative interpretation would be that the Skrydstrup Woman moved during her teenage years to an area with lower strontium isotopic values than the place from which she originated, an explanation that correlates well with the strontium isotope ratios from the hair segments.

Due to the lack of general large-scale bioavailable strontium baseline coverages over Europe it is at the moment difficult to pinpoint potential areas of origin for the Skrydstrup Woman. Furthermore, several areas within Europe might be characterized by overlapping ranges which provides additional difficulties and challenges in delineating specific potential areas of origin. At the moment, the closest areas that seem to meet baseline ranges characterized by ^87^Sr/^86^Sr values of ~ 0.713 are: to the south, middle-eastern Germany [55], northeastern Netherlands [56], and northeastern Czech Republic [57]; to the north, southern Sweden [58]; and to the west, some areas in Britain [59].

When the biological profile is combined with the strontium isotope data, we can reconstruct a detailed timeline of the Skrydstrup Woman’s movements. We are able to conclude that the Skrydstrup Woman migrated from a region outside present-day territorial Denmark to the Skrydstrup area when she was between 13 to 14 years old. After this displacement, she resided in the area around Skrydstrup for the rest of her life.

Furthermore, our incremental hair analyses using carbon and nitrogen stable light isotopes (S1 Text) allow us to obtain a diachronic picture of health and nutritional status for her final months of life. The average isotopic data yielded a δ^15^N = 8.5 ‰ and a δ^13^C = 22.4 ‰, reflecting a terrestrial diet broadly consistent with data on human hair from other Danish Bronze Age oak-coffin burials (S1 Text). The data trend as reflected by a sigmoidal curve for the δ^13^C isotope data (S1 Text) is also suggestive of seasonal variation in dietary consumption. However, these apparent variations are statistically not fully discernable as they lie within the analytical error determined from repeat measurements of internal and international standards (defined as ± 0.2 ‰ or better). The carbon to nitrogen atomic ratio (C:N) is a little higher than would be expected for modern human hair, with correspondingly negative δ^13^C values. This is likely to be due to contributions from the depositional environment (high tannic/ humic content) and/ or from residual effects stemming from the conservation treatment using glycerol applied post-excavation.

Interestingly, when we compare the trend data-sets of δ^13^C values for the Skrydstrup Woman (S1 Text) with those recently published for other Bronze Age humans, such as the Egtved Woman [5] and the Borum Eshøj C Woman, we note obvious similarities which suggest that these individuals could each have died at comparable annual time periods. This would support previous findings based on the examination of botanical remains in the Skrydstrup and Egtved burials which mature in the same annual season [60], namely in this case the summer months.

Because the sample for aDNA extraction was very small and thus yielded a very low DNA concentration, the library consequently required an excessive number of PCR-cycles to become sequencable. This resulted in two observations when the amplified and purified library was profiled on an Agilent Bioanalyzer 2100: 1) A very high concentration of adapter dimer, making it inefficient to sequence with Next Generation Sequencing technology and 2) PCR products in the negative extraction control with similar length distribution and concentration such as in the DNA library from the hair sample. For these reasons, we decided not to perform further aDNA analyses.

Finally, the microscopic investigations showed that the hair is fairly coarse with diameters of approximately 80–100 μm (measured while focused on the center of the strand). The hair appears light orange brown in transmitted light, which is most likely due to staining from the depositional environment. Regions on several of the hair segments were narrower and darker in color. These regions seem to be damaged, possibly by microbial attack. Carl M. Steenberg who examined the Skrydstrup Woman’s hair in the 1930s also postulated that some of the hair had been flattened by stones in the grave and that this compression was made easier because the hair was softened by the water flowing through the gravesite [10]. Nevertheless, the cuticle scale pattern is still evident on portions of the surfaces of all of the hairs examined. The hair sample taken furthest from the scalp has a clear defined and continuous medulla. Similarly, Carl M. Steenberg found medullae in most hairs fibers examined [10]. The segments taken from closer to Skrydstrup Woman’s head has an interrupted medulla, but it is not unusual for the proximal and distal ends of the hair fibers to vary in dimensions and potentially lack a medulla.

No discernable pigment granules were visible in most of the examined hair sections, probably due to the previously mentioned staining. Only one fiber displayed small dark spots across the cortex that could be pigment granules (S1 Text). That fiber was potentially missing the possible pigmentation which is present in about a quarter of its length. Although pigment granules have been found to be fairly durable in most burial environments, they can also degrade [61,62]. Importantly for our present investigation, the mound’s waterlogged, anoxic and acidic environment is known to have an impact on hair color, with less stable eumelanin pigmentation (responsible for black-brown coloration) subject to change relative to the more stable phaeomelanin (responsible for red-yellow coloration) [62]. Thus the more stable red-yellow coloration might mask the true original coloration. We are currently unable to more concretely elaborate on the original hair color of the Skrydstrup Woman.

When examined from an archaeological standpoint, this one-time movement of the Skrydstrup Woman (as revealed by the strontium isotope analyses of her teeth enamel and scalp hair), an elite female in her “age of marriageability”, might suggest that she migrated with the aim of establishing an alliance between chiefdoms rather than for trade purposes. These findings contrast with the recently published findings of another comparable (in relation to status, age and prehistoric Period) nonlocal Bronze Age female individual unearthed in Denmark, the “Egtved Woman”, which showed a very different mobility pattern of repeated travels during at least the last two years of her life [5]. Furthermore, the strontium isotope analyses of the Egtved Woman’s scalp hair, nail and tooth enamel were interpreted such that this individual spent less than a year, cumulatively, in present-day territorial Denmark over her life span. In contrast, the Skrydtstrup Woman probably resided in the Skrydstrup area for several years. In common with the results from the studies of the Egtved Woman, the grave goods interred with the Skrydstrup Woman, i.e. her gold spiral rings, garments and horn comb, do not explicitly suggest a nonlocal origin (S1 Text). This mirrors other European Bronze Age cemeteries, where grave goods do not reflect the origin of the individuals with whom they are buried [63,64]. Therefore, even though the Egtved and the Skrydstrup Women were both buried in burial mounds in present-day Denmark, in burial monuments which have long been considered to represent the resting places of the ancient local elites, strontium isotope results prove that these two Bronze Age Women originated from areas far away from where they were finally buried. Moreover, the different strontium isotope signatures in their first molars indicate different areas of origin for both of the two women. Finally, and most importantly, with regards to our current study project aiming at investigating the mobility of Bronze Age Women, strontium isotope data infer defined but very distinct and individual mobility patterns (S1 Text). These differences in mobility and migration patterns suggest that we should begin to think of a much more nuanced model for Bronze Age movement, which should consider motivation and patterns (repetitive versus one-time) of mobility. In this way, we can both refine existing models and create new avenues for future archaeological research.

## Conclusion

We present a novel set of investigations, in which high-resolution strontium isotope analyses of different human tissues of a single individual (representing complementary lifeways information) are combined with physical anthropological analyses (including CT scanning and 3D visualizations). When superimposed, they provide the first case study in which a migration timeline can be related to the chronological age of the individual that moved. Our study is based on the human remains of a Bronze Age female known as the Skrydstrup Woman who was discovered in Denmark. Our ^14^C dating place the Skrydstrup assemblage in the Nordic Early Bronze Age Period III (1300–1100 BC). Although the acidic environment within the oak-coffin, in which the Skrydstrup Woman was burried, unfortunately prevented the preservation of human aDNA, our incremental δ^13^C and δ^15^N isotope hair analyses reveal a terrestrial diet with some indications of seasonal variation and the microscopy analyses showed that she had fairly coarse hair. Furthermore, our anthropological analyses reveal that the Skrydstrup Woman was between 17–18 years old when she died, and that she moved from her place of origin -outside present-day territorial Denmark- to the Skrydstrup area between c. 47 to 42 months before she died. Hence, she was between 13 to 14 years old when she migrated to the Skrydstrup area in Denmark. After this displacement, she resided in the area around Skrydstrup for the rest of her life. We therefore hypothesize that this one-time movement of an elite female during her “age of marriageability” suggests that she migrated with the aim of establishing an alliance between chiefdoms, rather than being part of a trade visit. In contrast to previous studies on the Egtved Woman (another Bronze Age female) revealing repeated long-distance travels, the results presented herein on the Skrydstrup Woman point to a mobility pattern characterized by a single long-distance displacement.

The detailed investigations of Skrydstrup and the Egtved females indicate that young elite women from the Nordic Early Bronze Age might have had very different reasons for moving from one place to another, thereby adding yet another interesting layer of complexity to our understanding of the social dynamics of European Bronze Age society.

## Supporting information

S1 Text Archaeological contextualization and additional analytical details with diagrams and data tables. (PDF)
