# Supplementary material for: A matter of months: High precision migration chronology of a Bronze Age female
Source: PLoS One. 2017 Jun 5;12(6):e0178834. doi: 10.1371/journal.pone.0178834 (PMC5459461; doi:10.1371/journal.pone.0178834)
Supplement: S1 Text — (PDF) [file pone.0178834.s001.pdf]

Supplementary Information (S1 Text)

"A matter of months: High precision migration chronology of a Bronze Age female"

Karin Margarita Frei, Chiara Villa, Marie Louise Jørkov, Morten E. Allentoft, Flemming Kaul, Per Ethelberg, Samantha Scott Reiter, Andrew Wilson, Michelle Taube, Jesper Olsen, Niels Lynnerup, Eske Willerslev, Kristian Kristiansen and Robert Frei

The archaeological context of Skrydstrup's burial mound

The Skrydstrup Woman was unearthed in 1935 from a burial mound in southern Denmark (1). The mound from which she was recovered was part of a mound-group consisting of a total of eight burial mounds (2, 3). The mound-group is located southwest of the village of Vojens in a geographical and geological liminal zone that divides the landscape in two by the East-Jutlandic maximum glaciation line of the last Ice Age (Weichsel). This end-moraine line stretches from south to north and divides the landscape: to the west, the land is flat and sandy, whereas to the east the landscape is dominated by hills and easily recognizable moraines. In consequence, the central trade/exchange route (known in Danish as the "Hærvej") (4) ran parallel to this line, and hence also passed close to the Skrydstrup site.

Interesting for the archaeological context of the Skrydstrup Woman's burial is a contemporary settlement of longhouses nearby which were discovered in 1993. These longhouses are located

about 600 meters to the east of the burial site (3) and consist of three 3-aisled structures, which according to the ^{14}C-dates, are contemporary with the Skrydstrup Woman's mound-group and therefore potentially point to some kind of relationship. One of the houses (House IV) exhibit an impressive total ground area of approximately 500 m^2, making it the largest Early Bronze Age house excavated in Denmark to date. Another of the houses (House I) also stands out as ^{14}C-dates of the house overlap and are very similar to the ^{14}C-dates obtained for the Skrydstrup Woman. It might be possible, therefore, that the Skrydstrup Woman had some kind of knowledge about the existence (or even had a more direct relation) to this particular house (3).

In sum, the impressive size of the longhouse, combined with its proximity to the central trade route ("Hærvej") provide evidence of this area's important role in interregional trading networks during the Nordic Early Bronze Age (most specifically in Periods II and III, cf. (5).

The Skrydstrup Woman

The Skrydstrup woman's remains represent the mound's primary grave and consist of skeletal as well as soft tissues including parts of her cheeks and chin, eyebrows, eyelids, eyelashes and her long hair (1, 6). Although only the hair remains of the latter, a large part of Skrydstrup Woman's skeleton is still intact; the teeth in particular are well preserved (1).

Her hair (which is of particular interest for our present study) measures in at over 60 cm, and is set in a highly complex hairstyle (1). In order for her locks to be set, her hair was first combed forward and downwards over a hair pad before being bound by a wool cord. The hair was then plaited across the forehead from temple to temple before finally being covered with a hairnet that seems to have been made from horse hair (6).

Anthropology

Anthropological investigations revealed that the posterior portion of the skull had been crushed *post mortem* and there are only limited remains of the post-cranial skeleton. The left scapula is represented by a very small fragment of the glenoid, while the right scapula includes the glenoid, coracoid and part of the scapula's spine and lateral border. Only the right clavicle is present, but it is fragmented. From the left arm, a fragmented shaft of the ulna, a fragment of the distal radius, the scaphoid and lunate as well as fragments of the proximal 5th, 3rd and 2nd metacarpals are present. From the right arm, the shaft and distal end of the humerus, the shaft of the ulna and the distal third of the radius as well as the capitate and the 3rd to 5th metacarpals and an intermediate hand phalanx are present. In terms of the torso, a few rib fragments, the manubrium and sternum as well as 19 vertebral bodies are present (the lower four cervical, 11 thoracic and four lumbar vertebrae). The os coxae are present, although both right and left pubic symphyses were lost *post mortem* and the superior right ilium suffered some damage.

Although the right femur is present, the distal end is damaged. By contrast, the right tibia is present, but has a damaged proximal end. The right calcaneus and talus are present and so is the right navicular, lateral, intermediate and medial cuneiform as well as the first and second metacarpals.

The left femur is the best-preserved long bone of the Skrydstrup Woman. It is unfortunately mainly held together by the black glycerin coating mentioned in the main text. It has several fracture lines incurred *post mortem*. Due to *post mortem* damage prior to the glycerin coating, the distal one third of the bone is bent slightly in an anterior direction. The maximum length of the left femur was measured to be 469 mm, 0.6 mm less than the measurement from 1939 (1). The difference between

the two measurements could be due to a measurement error or due to the shrinkage of the bone over the last seven decades.

There is an ongoing debate as to which method should be applied in estimating living stature from archaeological skeletal remains (7). The most widely used method within the osteoarchaeological literature is that by Trotter (8). Although generally considered the most accurate, the equations were developed on modern American samples (American females from various social backgrounds who died in the 1960s). Unfortunately, the correlation between long bone length and living stature is not always reliable within a living population, let alone between modern populations and ancestral groups. Trotter's methods (8) are, therefore, unlikely to be truly applicable to Danish and/or European Bronze Age skeletal remains. For this reason, we decided to apply the methods developed by Ruff et al. (9) for Holocene European skeletal remains using the maximum length of the femur for females. This yielded a stature estimate of 169.7cm±2.92cm.

The length of the Skrydstrup female measured in the coffin (from the top of the skull to the heel) was *c*. 171.5-172 cm. The stature estimate using Ruff et al. (9) is clearly very similar. Still, because of possible secular shifts in body proportions we also considered comparing the length and proportions of the Skrydstrup Woman's femur to those of other contemporary females. Analyses of Bronze Age populations from central Europe show a marked variation in femur lengths and proportions for males and females (10). The Skrydstrup Woman's femur length is closer to the mean male femur length (mean 442.2-445.3 cm) than to the mean female femur length (mean 403.1cm to 411.4 cm), based on the central European Bronze Age dataset. This seems to indicate that Skrydstrup Woman was very likely a tall female for her time.

Due to Denmark's Late Bronze Age cremation tradition as well as the country's particular soil conditions, there are very few skeletal remains to which the Skrydstrup find can be compared to.

One of these rare points of comparison is that of the female oak coffin burial from Borum Eshøj C whose left femur is slightly shorter (421mm) and is also more robust than that of the Skrydstrup Woman, and also exhibits marked muscle attachments. Despite the fragmentation, it is clear that the Skrydstrup Woman's long bones are both unusually long and slender at the same time. Initial measurements were taken of the long bones as well as the cranium, irrespective of their poor preservation. Even then, osteologists acknowledged that any measurements they took would not be representative of the true lenghts had the bones been intact. The robusticity index of the left femur was initially calculated to be 10.9 (1). Unfortunately, re-measurement was prevented by the glycerin.

No evidence of pathology was found, although the Skrydstrup Woman did exhibit a congenital anomaly insofar as she had a missing styloid of the third left metacarpal. The latter, however, would have had no impact on either her health or daily activities.

CT scanning was performed both of the skull and the postcranial bones. The head was CT scanned in 2011 using a Siemens Sensation 4 using 1 mm slice thickness, 1 mm slice increment and a smooth reconstruction algorithm. Post cranial bones were CT scanned in 2016 using a Siemens Somatom Definition with 0.6 mm slice thickness, 0.4 mm slice increment and a sharp reconstruction algorithm. Post-processing of the images was done using Mimics (Materialise) and 3D visualization was created using the segmentation methods described by Lynnerup (11) and Villa & Lynnerup (12).

Stable isotope analyses ($\delta^{13}C$ and $\delta^{15}N$)

We conducted stable isotopes analyses of a 12 cm long sample of Skrydstrup Woman's scalp hair to investigate her dietary habits during roughly the final year of her life. The 12 cm long hair

sample was cut into a total of 8 segments (each measuring roughly c. 1.5 cm) to provide high resolution incremental data on the Skrydstrup Woman's diet.

The following tables and figures show values for incremental measurements ($\delta^{13}C$ and $\delta^{15}N$ ratios of hair) recorded for the Skrydstrup Woman on instrument run ACQ625.

Table 1. Isotopic results for samples of hair (bulk values, i.e. not sub-sampled)

Sample	Amt%N	Amt%C	$\delta^{15}N$	$\delta^{13}C$	n	C:N
1	10.8	41.2	8.80	-22.31	2	4.47
2	10.5	41.6	8.25	-22.55	1	4.61
3	11.6	45.0	8.27	-22.26	1	4.52
4	11.2	40.2	8.83	-22.43	1	4.17
		av.	8.54	-22.39		4.44

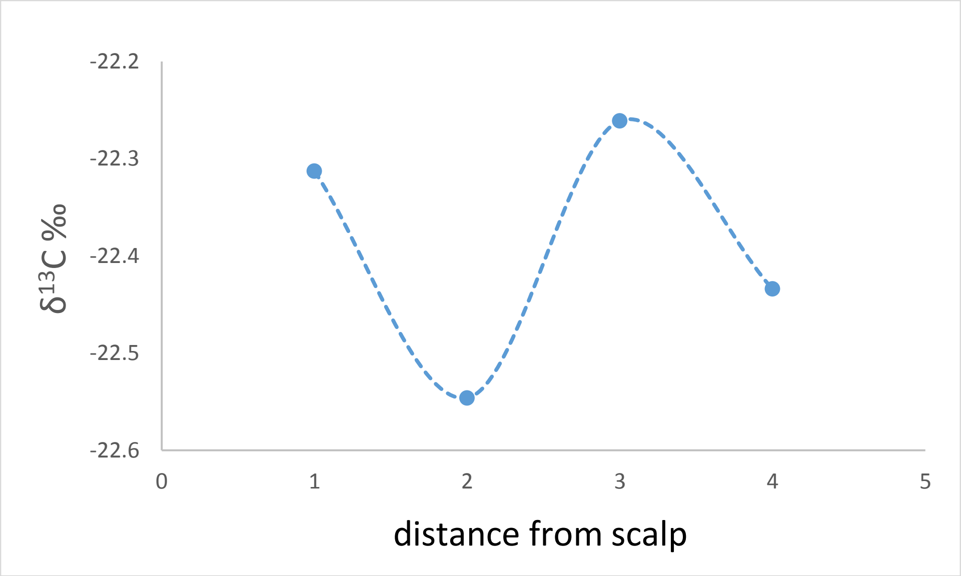

Figure 1. $\delta^{13}C$ values for samples of hair

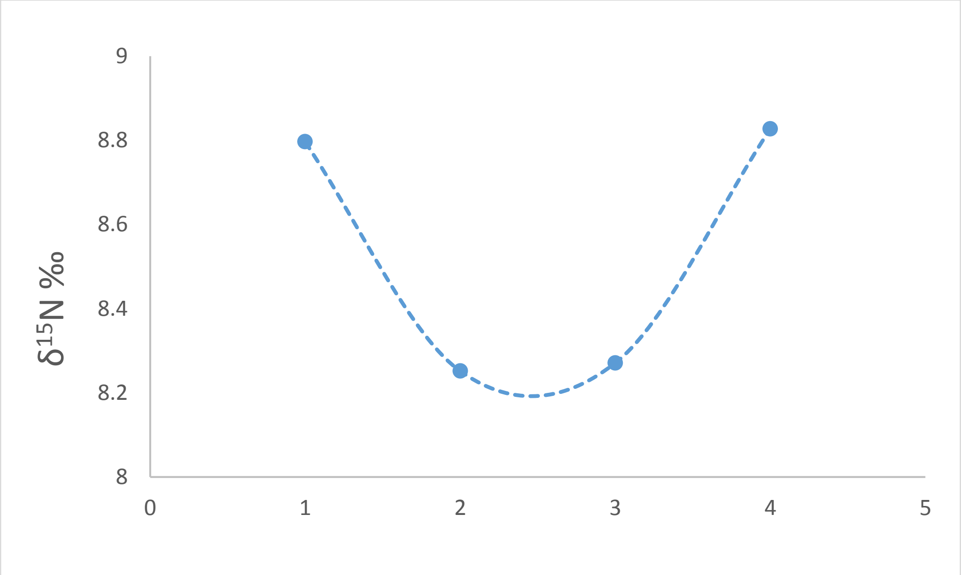

Figure 2. $\delta^{15}N$ values for samples of hair

Table 2. Incremental isotopic results for sub-samples of hair

Sample	Amt%N	Amt%C	$\delta^{15}N$	$\delta^{13}C$	n	C:N
1a	10.7	40.4	8.97	-22.25	1	4.42
1b	11.2	42.7	8.45	-22.61	1	4.46
2a	9.9	40.0	8.33	-22.86	1	4.73
2b	11.1	43.2	8.23	-22.31	1	4.52
3a	10.5	41.3	8.20	-22.40	1	4.60
3b	11.0	41.7	8.16	-22.09	1	4.40
4a	11.9	41.1	8.62	-21.96	1	4.04
4b	10.9	38.7	9.03	-22.28	1	4.14

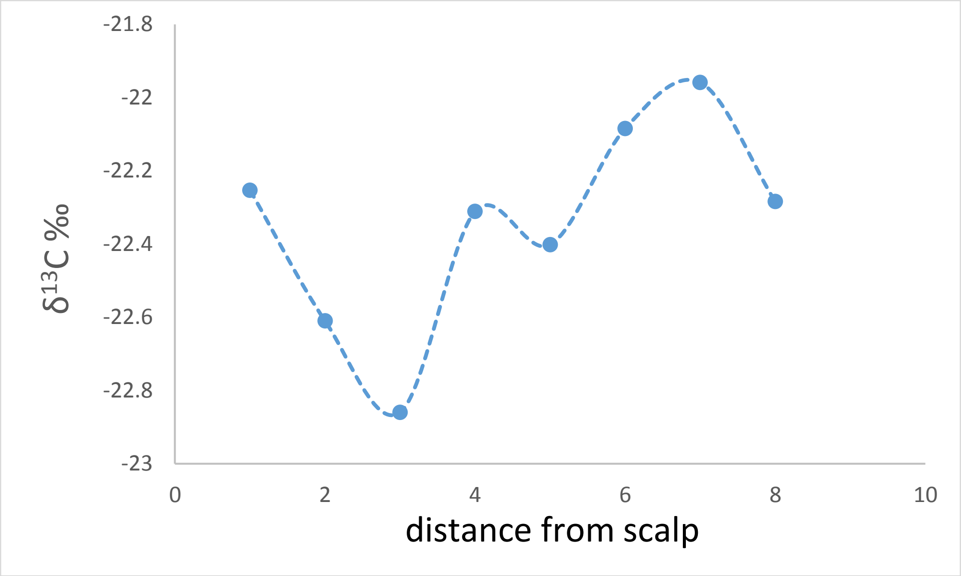

Figure 3. δ^{13}C values for sub-samples of hair.

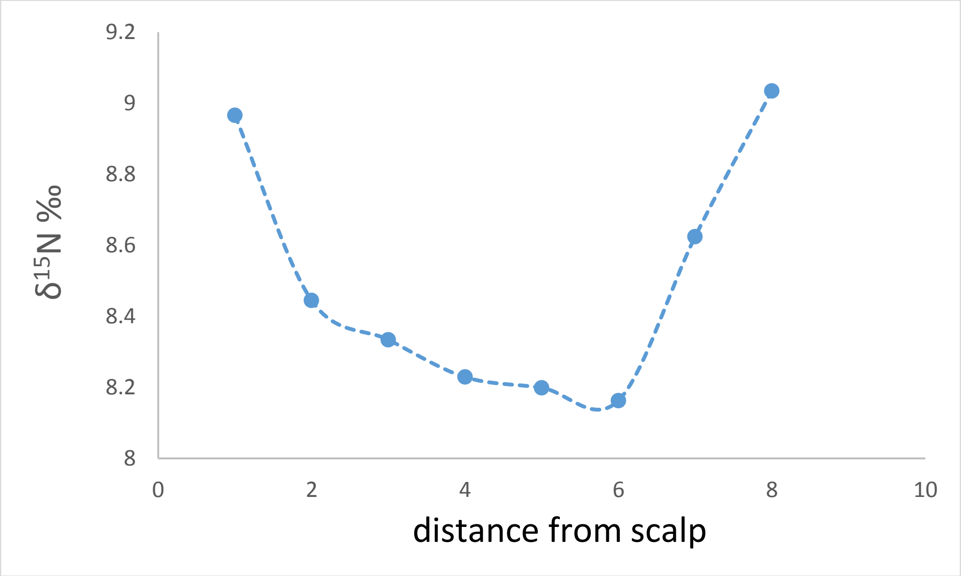

Figure 4. δ^{15}N values for sub-samples of hair

The results of our incremental hair analysis using carbon and nitrogen stable light isotopes are presented in Supp. Inf. Table 2. The aim was to obtain a diachronic picture of health and nutritional status for the final months of Skrydstrup Woman's life. The average δ^{15}N and δ^{13}C values

including all samples are calculated at $\delta^{15}N$ = 8.5 ‰ and $\delta^{13}C$ = 22.4 ‰, values which reflect a terrestrial diet broadly consistent with results from human hair of other Danish Bronze Age oak-coffin burials (13). In the following, we compare these values with other Danish Bronze Age coffin burials - to examine comparative data on nutrition/health in individuals from similar contexts. For example, previous studies showed that the Egtved Female returned mean values $\delta^{15}N$= 8.6 ‰ and $\delta^{13}C$= 21.6 ‰ (13), the Trindhøj Male is characterized by $\delta^{15}N$=9.5 ‰ and $\delta^{13}C$ =21.5 ‰, the Borum Eshøj A Male yielded $\delta^{15}N$= 8.9 ‰ and $\delta^{13}C$ = 21.5 ‰, and finally, the Borum Eshøj C Female yielded $\delta^{15}N$= 9.1 ‰ and $\delta^{13}C$= 21.1 ‰. The similarity of these average isotope values, including the values determined herein for the Skrydstrup Woman (Supp. Inf. Figs. 1 and 3) suggests that all of these individuals could have died at a similar time of year. This would coincide with previous observations which are based on the plant/flower remains in the graves of Skrydtstrup (a fully-developed cow parsley *Anthriscus silvestris*) (1) and Egtved (14). As pointed out by Bergerbrant (15), the plant remains in both burials indicate that the respective burials most probably took place between July and October when these plants become mature.

It is however more difficult to interpret the $\delta^{15}N$ values. $\delta^{15}N$ signatures may be influenced by seasonal dietary variation, access to dietary protein and by conditions of physiological stress, such as chronic illness, pregnancy, or nutritional stress. $\delta^{15}N$ data for the Skrydstrup Woman (Supp. Inf. Figs. 2 and 4) show an opposing trend when compared with the hair $\delta^{15}N$ values measured for the Egtved Woman, with a characteristic increase in $\delta^{15}N$ values in the final months of the Skrydstrup Woman's life.

Microscopic analyses

Several scalp hair fibers from the Skrydstrup Woman were examined with a stereo microscope and then a transmitted light microscope as described in the main text. Remnant cuticular scales were found in places on all of the hair fibers. Only one of the examined fragments showed signs of possible pigment granules (Supp. Inf. Fig. 5 below).

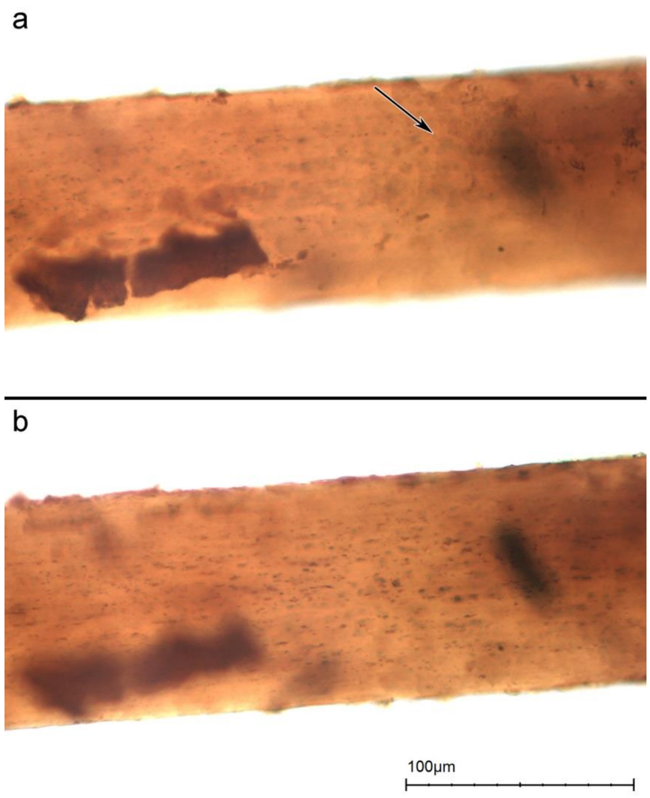

Figure 5. Transmitted light micrographs of a hair fiber taken from near the Skrydstrup Woman's neck in whole mount. The images were taken with the microscope focused at two depths: a) the upper surface of the hair showing a few remaining cuticular scales as well as some surface contamination and b) at the center of the strand showing an array of small dark spots in the cortex that may be pigment granules. (Plane polarized light, neutral density filter, embedded in polyethylene).

^{14}C analyses

We performed ^{14}C analyses by AMS of the Skrydstrup Woman's scalp hair, which revealed an age of 3009 ± 27 BP (Lab. nr. AAR: 25433). This age corresponds to the Nordic Early Bronze Age Period III (1300 -1100 BC).

Prior to ^{14}C analysis, the hair sample was deeply pre-cleaned by submerging the hair in a mixture of acetone and ethanol (on a hotplate) and subsequently rinsed with demineralized water. The C:N ratio of 3.0-3.8 measured after these treatments indicate that the pre-cleaning procedure was successful as it corresponds well with C:N scalp hair values of modern humans.

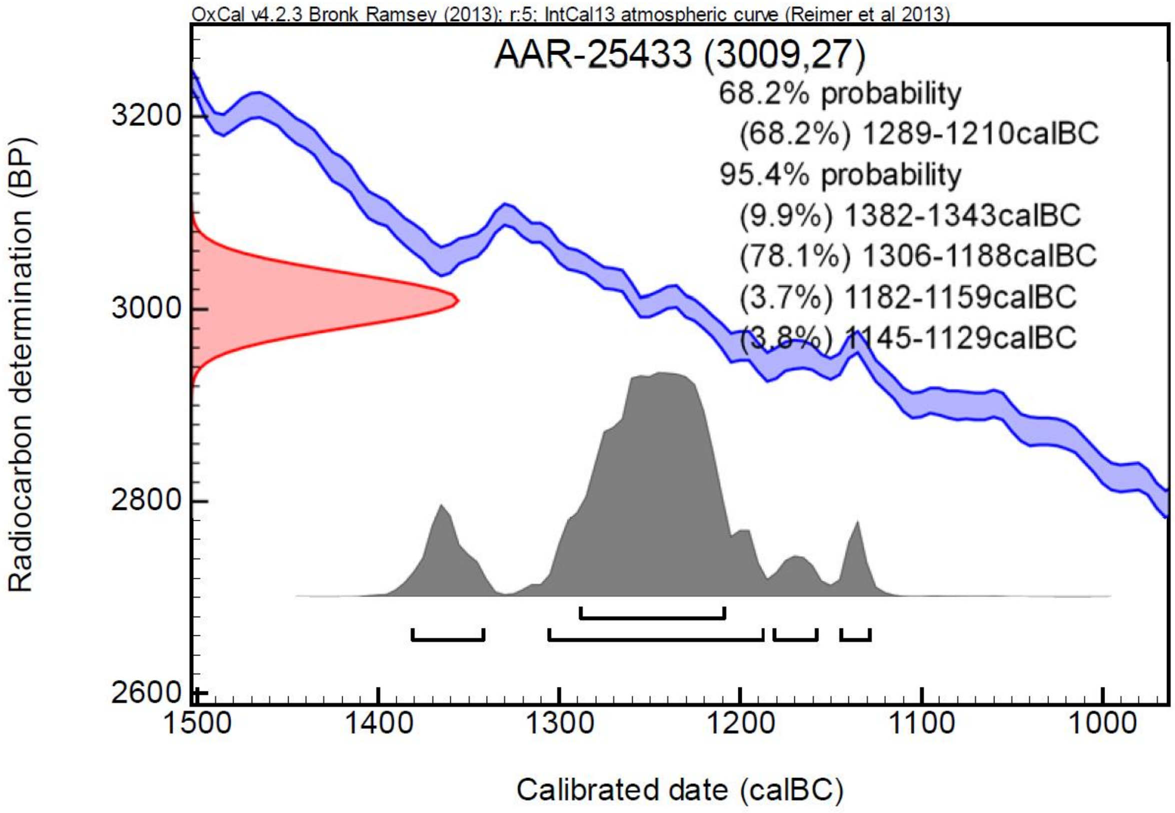

Figure 6. Radiocarbon dating of the Skrydstrup Woman's scalp hair.

The gold spiral rings

The strontium isotope analyses of the Skrydstrup Woman's first molar presented herein clearly indicate a nonlocal origin. However, it is difficult to pinpoint her geographical origin by means of those investigations alone. Therefore, we turn to the archaeological context in an examination of the grave goods for clues that might provide some indications of potential provenance regions. At least at first glance, the gold spiral rings seem to be the most nonlocal objects within Skrydstrup's grave goods. That being said, the earrings are relatively simple in manufacture: they are made of gold wire with a round profile wrapped into a series of loops (Fig. 3) which are often refered as 'endless double wire'.. The presence of various varieties of wire industries was present in Europe from the Early Bronze Age on through until late prehistory (16).

Although there are many examples of similar head jewelries recovered from the temple area of female inhumation graves from Early Bronze Age graveyards outside Denmark, such as those in Germany (17), Austria (18) Slovakia (19) and Poland (20), many of those examples are either made of bronze or, if made of gold, exhibit a much smaller loop diameter than the example from Skrydstrup.

There is an excellent corollary for the Skrydstrup earrings much closer to home: the rich female inhumation burial from Melhøj at Mjallerup (Aalborg, north Jutland, Denmark (21); (Supp. Info. Fig. 7). Excavated in 1930 by the National Museum of Denmark, the Melhøj burial mound contained a stone cist whose bottom was covered by wood (coffin remains) and which included animal hair, the badly-preserved remains of the deceased and a series of bronze artifacts which, when compared to the two spiral gold rings recovered on either side of the Skrydstrup Womans's skull (Fig. 3), are important and relevant for our current discussion about the Skrydstrup Woman's

origin. The placement in the grave, the material and the general form of the Melhøj spiral rings mirror those recovered from Skyrdstrup, however they are smaller in size. The Melhøj burial was richer than the one from Skrydstrup insofar as it included various bronze objects, including a tutulus belt disc, a tutulus, two bracelets, a necklace, a long pin, a small fibula, an ankle ring and a 28 cm-long dagger (21). The objects all place this burial in Nordic Bronze Age period III. Melhøj's organic material has been ^{14}C dated at 1220-980 BC (+/- 1 std.), which imply that the Melhøj and Skrydstrup burials belong to the same period, namely the Nordic Bronze Age Period III, and were probably almost contemporary (21). Indeed, the presence of an embroidered border among the textile fragments from Melhøj, which must undoubtedly be interpreted as the ornamentation of the neckline of a blouse similar to the one recovered from the Skrydstrup burial (21), serves as further evidence for the similarity of the burials at Skrydstrup and Melhøj.

That being said, the gold spiral rings from the Skrydstrup burial also find close correlations in the gold wire industry which blossomed in the Urnfield and Tumulus cultures of Bohemia. Particularly in eastern Bohemia (Czech Republic) well over 400 gold objects are recorded which sum up to a total gold weight of approximately 5 kg (16). Most of these gold finds are wire objects, dominantly of the double wire variety (22), as the one found with the Skrydstrup Woman. As is the case with the find from Skrydstrup, the Bohemian gold wire rings exhibit round or sub-round cross-sections and are twisted into a series of loops, often made of doubled wire. However, the closest central European parallels to the Skrydstrup find are seldom recovered from funerary contexts; they are more often recovered from hoard finds.

While there are similarities between the Skrydstrup spiral rings and other contemporary finds from e.g. Bohemia, the discovery of similar material and a similar usage of the gold spirals at Melhøj in

Denmark (supported by comparative ^{14}C dates and the presence of embroidered blouses), leaves us with no clear indication for the Skrydstrup Womans's origins.

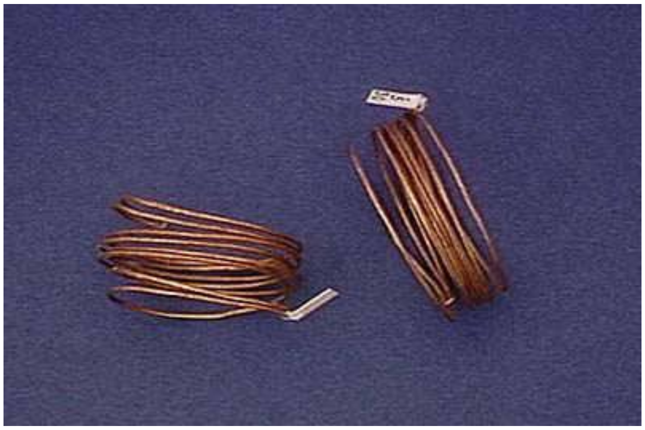

Figure 7. The gold spiral rings from the Melhøj Barrow, Nordic Bronze Age Period III. They seem to be typologically related to the Skrydstrup spiral rings (Photo: Courtesy of National Museum of Denmark).

References:

1. Broholm HC, Hald M. Skrydstrupfundet. København: Nordisk Forlag; 1939.
2. Aner E, Kersten K. Die Funde der älteren Bronzezeit des nordischen Kreises in Danemark, Schleswig-Holstein und Niedersachsen, Nordslesvig – Nord, Haderslev Amt. Neumünster: Wachholtz Verlag; 1984.
3. Ethelberg P. Bronzealderen. Det sønderjyske landbrugs historie Sten- og Bronzealder. Haderslev: Historiske Samfund for Sønderjylland; 2000.
4. Bang J. The Routh to a history oft he cultural landscape: a Danish record of prehistoric and historic roads, tracks and related structures. In: Bergerbrant S, Sabatini S, editors. Counterpoint: Essays in Archaeology and Heritage Studies in Honour of Professor Kristian Kristiansen. Oxford: Archaeopress; 2013. p. 703-15.
5. Johansen KL, Laursen ST, Holst MK. Spatial patterns of social organization in the Early Bronze Age of South Scandinavia. J Anthropol Archaeol. 2004;23:33–55.
6. Broholm HC, Hald M. Costumes of the Bronze Age in Denmark. Copenhagen and London1940.
7. Jørkov ML. Stature in 19th and early 20th century Copenhagen -A comparative study based on skeletal remains. Economics & Human Biology. 2015;19:13-26.
8. Trotter M, (1970). Estimation of stature from intact limb bones. In: Stewart TD, editor. Personal Identification in Mass Disasters. Washington: National Museum of Natural History; 1970. p. 71–84.
9. Ruff CB, Holt BM, Niskanen M, Sladék V, Berner M, Gerafalo E, Garvin HM, Hora M, Maijanen H, Niinimäki S, Salo K, Schuplerová E, Tompkins D. Stature and Body Mass Estimation From Skeletal Remains in the European Holocene. 2012;148:601-617.
10. Vančata V, Charvátová M. Post-Palaelithic Homo Sapiens evolution in central Europe: Changes in body size and proportions in the Neolithic and Early Bronze Age. Anthropologie. 2001; XXXIX/2-3:133-52.
11. Lynnerup N. Computed Tomography Scanning and Three-Dimensional Visualization of Mummies and Bog Bodies. In: Pinhasi R, Mays S, editors. Advances in Human Palaeopathology. Chichester, UK: Wiley; 2007.
12. Villa C, Lynnerup N. Hounsfield Units ranges in CT-scans of bog bodies and mummies. Anthropol Anz. 2012;69(2):127–45.
13. Frei KM, Mannering U, Kristiansen K, Allentoft ME, Wilson AS, Skals I, et al. Tracing the dynamic life story of a Bronze Age Female. Scientific Reports. 2015;5:10431.
14. Randsborg K, Nybo C. The Coffin and the Sun: Demography and Ideology in Scandinavian prehistory. Acta Archaeologica. 1986;55(161-184).
15. Bergerbrant S. Ordinary or extraordinary?, Redressing the problem of the Bronze Age corded skirt. Current Swedish Archaeology. 2014;22:73-96.
16. Hrala J. Überlegungen zum Gold in der mittleren und späten Bronzezeit in Böhmen und Mähren. In: Lehrberger G, Fridtrich J, Bebhard R, Hrala J, editors. Das Prähistorische Gold in Bayern, Böhmen und Mähren: Herkunft, Technologie, Funde: Památky Archeologické Supplementum; 1997. p. 169-89.
17. Meller H. Die neolithischen und bronzezeitlichen Goldfunde Mitteldeutschlands--Eine Übersich. In: Meller H, Risch R, Pernicka E, editors. Metalle der Macht--Frühes Gold und Silber. Saale2014. p. 611-716.
18. Neugebauer-Maresch C, Neugebauer J-W. Goldobjekte aus den Frühbronzezeitnekropolen Franzhausen I und II und Gemeinlebarn F. Mitteilungen der Anthropologischen Gesellschaft in Wien. 1988/89:101-43.

19. Bátora J. Das Gräberfeld von Jelšovce, Slowakei: Ein Beitrag zur Frühbronzezeit im Nordwestlichen Karpatenbecken. Kiel: Ötker; 2000.
20. Sarnowska W. Pierwsze zlote wyroby na Śląsku- First gold articles in Silesia. Silesia Antiqua. 1966;8:7-28.
21. Bender Jørgensen L, Munksgaard E, Stærmose Nielsen K. Melhøj-fundet. En hidtil upåagtet parallel til Skrydstrup-fundet. Aarbøger for Nordisk Oldkyndighed og Historie 1982. 1984:19-57.
22. Hrala J. Metal Artefacts and Artefacts of other Materials. In: Hrala J, Šumberová R, Vávra M, editors. Velim: A Bronze Age Fortified Settlement in Bohemia. Prague: Institute of Archaeology, Academy of Sciences of the Czech Republic; 2000. p. 219-56.
